# Can Asymptomatic or Non-Severe SARS-CoV-2 Infection Cause Medium-Term Pulmonary Sequelae in Children?

**DOI:** 10.3389/fped.2021.621019

**Published:** 2021-05-13

**Authors:** Ilaria Bottino, Maria F. Patria, Gregorio P. Milani, Carlo Agostoni, Paola Marchisio, Mara Lelii, Marco Alberzoni, Laura Dell'Era, Massimo L. Castellazzi, Laura Senatore, Barbara Madini, Maria C. Pensabene, Alessia Rocchi

**Affiliations:** ^1^Department of Clinical Sciences and Community Health, Università degli Studi di Milano, Milan, Italy; ^2^Pediatric Highly Intensive Care Unit, Fondazione IRCCS Ca' Granda Ospedale Maggiore Policlinico, Milan, Italy; ^3^Pediatric Emergency Department, Fondazione IRCCS Ca' Granda Ospedale Maggiore Policlinico, Milan, Italy; ^4^Pediatric Intermediate Care Unit, Fondazione IRCCS Ca' Granda Ospedale Maggiore Policlinico, Milan, Italy

**Keywords:** COVID-19, children, follow-up, complications, pulmonary function, ultrasound, long COVID, antibodies

## Abstract

Pulmonary complications in adults who recovered from severe acute respiratory syndrome coronavirus 2 (SARS-CoV-2) have been reported even in minimally symptomatic patients. In this study, lung ultrasound (LUS) findings and pulmonary function of children who recovered from an asymptomatic or mildly symptomatic SARS-CoV-2 infection were evaluated. We prospectively followed up for at least 30 days patients younger than 18 years who recovered from SARS-CoV-2 infection at the Fondazione IRCCS Ca' Granda Ospedale Maggiore Policlinico, Milan (Italy). All enrolled patients underwent LUS. Airway resistance measured by the interrupter technique test was assessed in subjects aged 4–6 years, whereas forced spirometry and measurement of diffusing capacity of the lungs for carbon monoxide were performed in subjects older than 6 years. To evaluate a possible correlation between pulmonary alterations and immune response to SARS-CoV-2, two semiquantitative enzyme immune assays were used. We enrolled 16 out of 23 eligible children. The median age of enrolled subjects was 7.5 (0.5–10.5) years, with a male to female ratio of 1.7. No subject presented any abnormality on LUS, airway resistance test, forced spirometry, and diffusing capacity of the lungs for carbon monoxide. On the other hand, all subjects presented Ig G against SARS-CoV-2. In contrast in adults, we did not detect any pulmonary complications in our cohort. These preliminary observations suggest that children with an asymptomatic or mildly symptomatic SARS-CoV-2 infection might be less prone to develop pulmonary complications than adults.

## Introduction

Both adults and children can be infected by the novel severe acute respiratory syndrome coronavirus 2 (SARS-CoV-2). However, a lower respiratory tract involvement has been observed mainly in adult patients (1), and pulmonary complications have been reported after recovery (2) even in many minimally symptomatic patients (3). Compared with adults, most children infected with SARS-CoV-2 have a milder clinical course (4). No data are currently available on pulmonary complications in children who recovered from non-severe forms of SARS-CoV-2 infection. The aim of this preliminary study was to evaluate lung ultrasound (LUS) findings and pulmonary function in children who recovered from an asymptomatic or mildly symptomatic SARS-CoV-2 infection.

## Methods

We prospectively followed up for at least 30 days after recovery patients younger than 18 years with a positive test for SARS-CoV-2 RNA (nasopharyngeal aspirate or pharyngeal swab) at the Fondazione IRCCS Ca' Granda Ospedale Maggiore Policlinico, Milan, Italy. Patients were considered recovered from infection after two negative nasopharyngeal aspirates (in subjects aged <6 years) or nasopharyngeal swabs (in subjects aged ≥6 years), performed at least 24 h apart. Exclusion criteria were the need for mechanical ventilation during SARS-CoV-2 infection, severe neurological impairment, a known pre-existing chronic respiratory disease (e.g., asthma), and a hemodynamically unstable cardiovascular disease. Subjects who developed a multisystem inflammatory syndrome were also excluded. All enrolled patients underwent a LUS. Airway resistance measured by the interrupter technique (Rint) test was performed in subjects aged 4–6 years. Forced spirometry and measurement of diffusing capacity of the lungs for carbon monoxide (DLCO) were performed in subjects older than 6 years.

A high-frequency (7–11 MHz) linear probe (SonoScape, S22 model) was used to perform LUS. When needed, techniques to minimize anxiety were adopted according to the age of the patients. The ultrasound was performed by an expert (>10 years of experience in point-of-care LUS) pediatrician (AR) while children were sitting (for posterior and anterior scans) and supine (for anterior scans) according to a pre-defined, systematic approach (5, 6). Both longitudinal and transversal sections were obtained, and six zones for each lung were separately considered. Anatomical lines (parasternal, mid-clavicular, anterior axillary, mid-axillary, posterior axillary, mid-scapular, and paravertebral) were used to visualize the different zones. The LUS score (LUSS) (7), a semiquantitative score (ranging from 0 to 3), was calculated. The score reflects the lung aeration loss caused by different lung tissue modifications. A LUSS equal to 0, i.e., the presence of A-lines and few non-confluent B-lines, was considered as normal (8). A score ≥ 1 or the direct detection of abnormal structures such as consolidations were considered pathological. All the pulmonary function tests were performed according to the ATS-ERS guidelines (9), by means of a validated spirometer (Vintus Body, Vyaire Medical). For the Rint-test, the participants were instructed to breathe normally through the mouthpiece and to keep the tongue against the floor of the mouth as recommended (10). At least five measurements were made during exhalation, and the median value was recorded. Hence, a post-bronchodilator assessment of airways resistance was performed 15 min after salbutamol (200 μg) administration.

Forced spirometry was performed in children sitting comfortably in a chair. After three tidal breaths, children were asked to perform a maximal inspiration and, immediately after, a forced, quick, complete exhalation for at least 6 s. Forced vital capacity (FVC), forced expiratory capacity in the first second of exhalation (FEV1), and the mean forced expiratory flow between 25 and 75% of the FVC (MEF 25/75) were measured on a minimum of three acceptable maneuvers. At least three maneuvers were repeated 15 min after the inhalation of salbutamol (400 μg) through a disposable spacer (11). The best values for each measurement were retained.

DLCO was measured by means of the single-breath test. Children were instructed to breathe at tidal volume three to five times and then to perform an unforced exhalation to residual volume. Then, a deep and quick inspiration of a gaseous mixture (0.3% of methane and 0.3% of CO) was followed by 10-s apnea and, finally, by a quick exhalation. Values of DLCO were expressed as percentages of reference values (12), corrected for alveolar volume and hemoglobin. The following respiratory findings were considered as abnormal: (1) a basal airway resistance decrease by 30% or more at Rint-test (13); (2) basal values of the FVC, FEV1, and DLCO <80% and of MEF 25–75 <70% of the expected values for the spirometry test; and (3) FEV1 improvement ≥200 ml or ≥12% after a bronchodilator (14).

To explore a possible correlation between pulmonary alterations and immune response to SARS-CoV-2, two semiquantitative enzyme immune assays were used, the Liaison SARS-CoV-2 S1/S2 IgG assay (DiaSorin, Italy) and the Elecsys Anti-SARS-CoV-2 assay (Roche, USA), which detect IgG antibodies against S1/S2 antigens and total Ig against the nucleocapsid of SARS-CoV-2, respectively. All tests and laboratory analyses were performed on the same day. Data are presented as median and interquartile range (IQR) or frequency and percentage for continuous and categorical data, accordingly.

The study was approved by the Institutional Ethical Committee (Comitato Etico Area 2, Milano, Italy, Approval Number 568_2020). All parents or legal guardians of the enrolled subjects signed a written consent form. Subjects aged ≥12 years gave their consent to participate.

## Results

We recruited a total of 16 out of 23 eligible children, between August 1 and September 30, 2020. Two of the seven remaining children were not enrolled because they lived far from Milan and it was not possible for them to return to the hospital, while the other five declined to participate. The median age of enrolled subjects was 7.5 (0.5–10.5) years, with a male to female ratio of 1.7. The median time from the diagnosis to the recovery was 37 (27–62) days. The median time from recovery to the follow-up tests was 67 (49–91) days. The clinical presentation at the diagnosis of SARS-CoV-2 infection is reported in Table 1. All patients were free of respiratory symptoms at the follow-up evaluation. All the 16 children presented A-lines at LUS in all pulmonary zones. Twelve had a homogenous A-lines pattern without B-lines. Three subjects had ≤2 non-confluent B lines in the posterior scans, and one had ≤2 non-confluent B lines in the anterior and posterior scans, indicating normal LUS findings with a LUSS equal to zero (Table 2). Basal and post-bronchodilator findings of Rint, spirometry, and DLCO tests are given in Table 3. Rint-test was performed on three patients. The values before the administration of salbutamol were rather high, as often observed in children. On the other hand, the variation between pre- and post-bronchodilator values were between +2 and −18%, pointing out normal airway resistances in all three subjects. Spirometry and DLCO were performed in seven patients, and in none of them, values < 80% of predicted were found.

**Table 1:** Baseline characteristics of the 16 included patients.

**Characteristic**	
*N*	16
Gender, female	6 (38)
Age, years	7.5 (0.5–10.5)
Non-caucasian	5 (32)
Clinical presentation	
Asymptomatic	2 (13)
Isolated fever	2 (13)
Fever and respiratory symptoms	2 (13)
Fever and gastrointestinal symptoms	2 (13)
Febrile seizure	3 (19)
Incomplete Kawasaki disease	1 (6.3)
Respiratory symptoms	3 (19)
Gastrointestinal symptoms	1 (6)
Length of hospital stay, days	4 (1–6)
Time to recovery, days	37 (27–62)
Time from recovery to enrollment, days	67 (49–91)

*Data are given as median and interquartile range or absolute values and percentages*.

**Table 2:** Description of the parenchymal and pleural lung findings at lung ultrasound.

**Lung ultrasound findings**	***N* (%)**
**Parenchymal findings**	
A-lines	16 (100)
B-lines ≤ 2 per zone	4 (25)
B-lines > 2 per zone	0 (0)
Confluent B lines/subpleural consolidation	0 (0)
Large consolidations	0 (0)
**Pleural findings**	
Smooth tine pleural line	16 (100)
Irregular/thickened pleura	0 (0)
Pleural effusion	0 (0)

*Data are given as absolute and relative frequency*.

**Table 3:** Pulmonary function tests of the enrolled children.

**Lung function test**	**Basal values**	**Post-bronchodilator values**	**% Variation**
**Forced spirometry**			
FEV1%	96 (94–102)	95 (86–106)	+0 (−4 to +2)
FVC%	95 (87–100)	97 (88–98)	−3 (−6 to +0)
FEV1/FVC	92 (87–97)	94 (90–98)	+2 (+1 to +3)
MEF 25–75%	105 (83–118)	108 (96–113)	+2 (−19 to +12)
**DLCO**	119 (111–132)		
**Rint**	180, 252, 258	147, 209, 264	−18, −17, +2

*Data are presented as median and interquartile range or absolute values and percentages*.

*FEV1, forced vital capacity in the first second; FVC, forced vital capacity; MEF 25–75%, mean forced expiratory flow between 25 and 75% of the FVC; DLCO, diffusing capacity of the lungs for carbon monoxide; RINT, airway resistance measured by the interrupter technique*.

All patients tested positive for SARS-CoV-2 antibody tests in both the described methodologies.

## Discussion

A high percentage of adults who recovered from SARS-CoV-2 infection presents with pulmonary dysfunctions even months after the infection (15). To the best of our knowledge, this is the first study investigating the medium-term effects on pulmonary function of SARS-CoV-2 infection within a pediatric population with typical SARS-CoV-2 respiratory symptoms. We did not detect any pulmonary complication or abnormality in our cohort, as opposed to adults.

Studies in adults have shown that ultrasound LUS and CT scan have a good correlation in the diagnosis of COVID-19 pneumonia (16). Also in children, the useful of LUS has been reported both for the initial workup of children affected by SARS-CoV-2 (17, 18) and for their lung monitoring during the infection (18). Although we could not compare the results of LUS with CT scans, we did not observe discrepancies among LUS findings, clinical findings, and other pulmonary tests. Taken together, the available data and the results of this preliminary study support the use of LUS both in the acute phase of SARS-CoV-2 infection and during the follow-up. Future studies should further evaluate the use of sonography in children with both asymptomatic and symptomatic SARS-CoV-2 infection.

In adults, long-term pulmonary sequelae after a mild SARS-CoV-2 infection might be the result of an abnormal inflammatory response (19). We speculate that children with an asymptomatic or mildly symptomatic SARS-CoV-2 infection might present with a less vigorous but more specific immune response than adults and be less prone to develop pulmonary complications (20–22). Noteworthy, all our patients tested positive for SARS-CoV-2 Ig G. The latter data are consistent with recently published studies showing that most children affected by SARS-CoV-2 develop specific IgG against the virus (23, 24). On the other hand, emerging evidence suggests that also children might develop the so-called “long Covid syndrome” (25). A study including 129 children with a SARS-CoV-2 infection found that few children (<5%) still present respiratory symptoms, such as pain and chest tightness, at 120 days of follow-up (26). Since the sample size of this study was limited, we cannot rule out that medium-term pulmonary complications might develop in a minority of children with mild or asymptomatic SARS-CoV-2 infection.

Further limitations of this study should be acknowledged. First, since patients presented only with a mild or asymptomatic infection, the present findings cannot be generalizable to all children who recovered from a SARS-CoV-2 infection, especially those affected by the pediatric multisystem inflammatory syndrome or by the new emerging variants. Second, we did not have data on LUS and pulmonary function at baseline. Third, since children were all in good clinical conditions and without apparent pulmonary abnormalities, CT scans were not performed during the follow-up evaluation. Finally, the length of follow-up was short, and the possible development of pulmonary long-term sequelae cannot be ruled out.

In conclusion, these preliminary observations suggest that children with an asymptomatic or mild infection do not develop pulmonary sequelae in the medium-term period. Larger studies with longer follow-up are needed to confirm these findings.

## Data Availability Statement

Data are available at the corresponding author upon reasonable request.

## Ethics Statement

The studies involving human participants were reviewed and approved by Ethical Committee of the Hospital (Comitato etico Milano area 2 parere 568_2020). Written informed consent to participate in this study was provided by the participants' legal guardian/next of kin. Written informed consent was obtained from the individual(s), and minor(s)' legal guardian/next of kin, for the publication of any potentially identifiable images or data included in this article.

## Author Contributions

IB, MFP, GPM, CA, PM, and AR: study conception and data interpretation. ML, MA, LD, MC, LS, BM, and MCP: methodology and data acquisition. IB, MFP, GPM, and AR: writing the first draft. CA, PM, ML, MA, LD, MC, LS, BM, and MCP: reviewing the draft. All authors approved the study as submitted.

## Conflict of Interest

The authors declare that the research was conducted in the absence of any commercial or financial relationships that could be construed as a potential conflict of interest.
